# Black race, sex, and extrapulmonary tuberculosis risk: an observational study

**DOI:** 10.1186/1471-2334-10-16

**Published:** 2010-01-22

**Authors:** Christina T Fiske, Marie R Griffin, Holt Erin, Jon Warkentin, Kaltenbach Lisa, Patrick G Arbogast, Timothy R Sterling

**Affiliations:** 1 Division of Infectious Diseases, Department of Medicine, Vanderbilt University Medical Center, Nashville, TN, USA; 2 Departments of Preventive Medicine and Medicine, Vanderbilt University Medical Center, Nashville, TN, USA; 3 Tennessee Department of Health, Nashville, TN, USA; 4 Department of Biostatistics, Vanderbilt University Medical Center, Nashville, TN, USA; 5 Center for Health Services Research, Department of Medicine, Vanderbilt University Medical Center, Nashville, TN, USA

## Abstract

**Background:**

Extrapulmonary tuberculosis is likely a marker of underlying immune compromise. Our objective was to determine race and sex differences in extrapulmonary tuberculosis risk in order to identify the optimal population in which to assess for host factors associated with extrapulmonary tuberculosis.

**Methods:**

We performed an observational study of all tuberculosis cases reported to the Tennessee Department of Health, January 1, 2000 to December 31, 2006. We compared the incidence of extrapulmonary tuberculosis by race and sex. We also examined risk factors associated with extrapulmonary disease among all persons with tuberculosis.

**Results:**

Extrapulmonary tuberculosis incidence per 100,000 population was 5.93 in black men, 3.21 in black women, 1.01 in non-black men, and 0.58 in non-black women. Among those with tuberculosis, black women were most likely to have extrapulmonary disease (38.6%), followed by black men (28.1%), non-black women (24.6%) and non-black men (21.1%). In multivariate logistic regression among persons with tuberculosis, black women (OR 1.82 (95% CI 1.24-2.65), p = 0.002), black men (OR 1.54 (95% CI 1.13-2.09, p = 0.006), foreign birth (OR 1.55 (95% CI 1.12-2.14), p = 0.009), and HIV infection (OR 1.45 (95% CI 0.99-2.11), p = 0.06) were associated with extrapulmonary tuberculosis.

**Conclusions:**

Black men and black women had the highest incidence of extrapulmonary tuberculosis, and high odds of extrapulmonary disease among persons with tuberculosis. These data suggest that factors in addition to tuberculosis exposure contribute to extrapulmonary tuberculosis risk in blacks.

## Background

The global burden of tuberculosis is enormous, with 9.2 million new cases and approximately 2 million deaths in 2006 [1]. However, most persons infected with *Mycobacterium tuberculosis *do not develop active tuberculosis. Identification of the risk factors that predispose to tuberculosis would allow for targeted strategies to prevent it and decrease its global burden.

Tuberculosis presents in a variety of clinical manifestations, but the pathogenesis that determines site of disease is not well understood. Extrapulmonary tuberculosis is likely a marker of underlying immune compromise. HIV infection is associated with an increased risk of extrapulmonary tuberculosis, and the risk increases as the CD4+ lymphocyte count declines [2]. Extrapulmonary tuberculosis is more common in children than adults, presumably due to an immature immune system in children [3]. We have previously noted subtle immunologic defects, such as low numbers of CD4+ lymphocytes and low unstimulated cytokine production, in HIV-seronegative adults with previous extrapulmonary tuberculosis [4,5]. Persons with extrapulmonary tuberculosis represent a population in which to evaluate immune defects that predispose to tuberculosis.

Epidemiologic studies have found that among persons with tuberculosis, blacks and women have a higher proportion of extrapulmonary disease compared with non-blacks and men, respectively [6,7]. These race and sex differences have been postulated to result from discrepancies in access to care, transmission dynamics, and underlying host co-morbidities[8] but the specific etiology remains largely unexplained. An assessment of a possible interaction between race and sex and extrapulmonary tuberculosis risk has not been evaluated in more than 30 years [9]. In addition, the race and sex-specific incidence of extrapulmonary tuberculosis, which is a better estimate of risk than the proportion of persons with extrapulmonary disease among persons with active tuberculosis, has also not been determined in a U.S. population in over 30 years[10]. Examining both of these parameters could help explain observed differences in tuberculosis risk between patient populations.

The objective of this study was to estimate race- and sex-specific incidence rates of extrapulmonary tuberculosis in Tennessee to identify the optimal population in which to assess host factors associated with extrapulmonary tuberculosis. We also analyzed race and sex differences in the proportion of extrapulmonary disease among persons who developed tuberculosis. In addition, we assessed for other risk factors for extrapulmonary tuberculosis in our population, such as age, foreign birth, and alcohol and drug use.

## Methods

### Study population

We identified all tuberculosis cases reported to the Tennessee Department of Health between January 1, 2000 and December 31, 2006. Demographic characteristics of Tennessee and details regarding the organization of the Tennessee Department of Health are provided in Additional file 1: Appendix-Tennessee Demographics. Previous studies, including one performed in Tennessee, have found reporting of tuberculosis cases to local and state health departments to be accurate [11,12]. Clinical and demographic data were obtained when patients presented for tuberculosis treatment.

The study protocol was approved by the Institutional Review Boards of the Tennessee Department of Health, the Nashville Metro Public Health Department, and Vanderbilt University Medical Center.

### Study definitions

Verified tuberculosis cases were grouped into three categories based on site of disease: pulmonary, extrapulmonary, or both. For the purposes of our analysis, patients with both pulmonary and extrapulmonary tuberculosis were classified as extrapulmonary. In Tennessee, four criteria are used to verify tuberculosis cases as defined by the Centers of Disease Control and Prevention: (1) isolation of *M. tuberculosis *from a clinical specimen, (2) a positive stain for acid-fast bacilli within a clinical specimen (3) clinical diagnosis, or (4) provider diagnosis [13]. A clinically verified tuberculosis case met *all *of the following criteria: a history consistent with tuberculosis, abnormal imaging, a positive tuberculin skin test, signs and symptoms compatible with tuberculosis, and treatment with two or more anti-tuberculosis medications. A case verified by provider diagnosis met the same criteria as a clinical diagnosis, but lacked a positive tuberculin skin test.

Per health department protocol, upon being identified as a tuberculosis case or suspect an individual is interviewed by health department staff and demographic, clinical, laboratory, and risk factor information is collected and subsequently reported to the Centers for Disease Control and Prevention. Race was classified for the purposes of this study as black and non-black (Caucasian, Asian, American Indian, Hawaiian/Pacific Islander). Substance abuse, including injection drug use, non-injection drug use, and excess alcohol use during the year prior to tuberculosis diagnosis were recorded during the initial patient interview. Alcohol consumption was recorded as excessive based on the judgment of tuberculosis clinic personnel at the time of interview.

### Statistical analysis

Tuberculosis incidence was calculated by dividing the number of tuberculosis cases during the study period by cumulative annual estimates of the mid-year Tennessee population 2000-2006 based on United States census data [14]. Annual census data were used to estimate person-time for rate calculations. Annual incidence rates were reported per 100,000 population, and stratified by age, sex, and race. Unadjusted rate ratios and 95% confidence intervals were calculated. We stratified these parameters by race and sex given the possibility of an interaction between these two variables.

Differences in categorical risk factors between persons with pulmonary and extrapulmonary disease were compared using Pearson's chi-square test. Differences in continuous risk factors were compared using the Wilcoxon rank-sum test. All risk factors of interest were determined *a priori *and included in an adjusted logistic regression analysis to determine those risk factors associated with extrapulmonary disease among persons with tuberculosis. In addition to an analysis which included only those patients with known HIV status, we imputed HIV status for persons in whom it was unknown and refit the regression model. Missing HIV status was estimated using a single imputation model [15]. To account for the additional variability of using an imputed risk factor, we used bootstrapping to estimate standard errors [16]. All analyses were performed using STATA software, version 10 (Statacorp LP, College Station, TX).

## Results

### Characteristics of the study population

There were 2,142 verified tuberculosis cases reported to the Tennessee Department of Health during the study period. Tuberculosis cases met the following verification criteria: 1693 persons (79%) had positive culture for *M. tuberculosis*; 3 persons (0.1%) had positive acid-fast staining of sputum or tissue; 344 cases (16%) were clinically verified; and 102 persons (5%) were verified by provider diagnosis.

There were 564 (26.3%) persons with extrapulmonary disease and 1577 (73.6%) with pulmonary disease. There was a higher proportion of black, HIV-infected, and foreign-born persons with extrapulmonary disease compared to those with pulmonary disease (Table 1). The most common sites of extrapulmonary TB were lymphatic and pleural (Table 2). Extrapulmonary disease was less likely to be culture-confirmed (64.4%) than pulmonary disease (84.3%) (p < 0.001). There was no detectable difference in culture-confirmed extrapulmonary tuberculosis between men and women (65.2% vs. 63%, p = 0.59) and non-blacks and blacks (64.3% vs. 64%, p = 0.94).

**Table 1:** Demographic characteristics of tuberculosis cases in Tennessee, 2000-2006

Cohort Characteristics	All TB cases (n=2142)^a^	Pulmonary TB (n=1577)	Extrapulmonary TB (n=564)	p-value^b^
Median age - years (IQR)	48.6 (32.8,67.8)	50.1 (35.6,69)	43.3 (27.9,64.5)	< 0.001

Male sex	1433 (66.9)	1084 (68.7)	348 (61.7)	0.002

Black race	934 (44.1)	639 (41.0)	294 (52.8)	< 0.001

Foreign born	389 (18.2)	262 (16.6)	127 (22.5)	0.002

HIV infection	195 (9.1)	134 (11.5)	61 (15.0)	0.071

Homeless	196 (9.2)	157 (10)	39 (6.9)	0.03

Injection drug use	37 (1.8)	27 (1.7)	10 (1.8)	0.93

Non-Injection drug use	216 (10.3)	164 (10.6)	52 (9.4)	0.40

Excess alcohol use	430 (20.3)	362 (23.2)	68 (12.2)	< 0.001

**NOTE**. Data are presented as number(%) except as noted. IQR, interquartile range.

^a^One case did not have site of disease(pulmonary or extrapulmonary) identified.

^b^Chi-Square or rank-sum test for pulmonary TB v. extrapulmonary TB.

**Table 2:** Distribution of extrapulmonary tuberculosis cases by site of disease

	Number of persons (%)
Extrapulmonary^a^	564 (100.0)

Lymphatic^b^	183 (32.4)

Pleural	153 (27.1)

Bone	63 (11.2)

Genitourinary	31 (5.5)

Miliary	31 (5.5)

Meningeal	24 (4.2)

Peritoneal	17 (3.0)

Other	78 (13.8)

^a^Extrapulmonary = extrapulmonary only *or *extrapulmonary + pulmonary

^b^Proportion of extrapulmonary cases

Of those with tuberculosis, 195 (9.1%) were HIV-infected, 1,376 (64.2%) were HIV-uninfected, and 571 (26.7%) had unknown HIV status. Persons with unknown HIV status were more likely to be female (57%), have a higher median age (66.4 years), identify themselves as white (66%), have been born in the United States (89%), and less likely to be homeless (4%) or use illicit drugs (2%).

### Incidence of extrapulmonary tuberculosis

**Figure 1:**
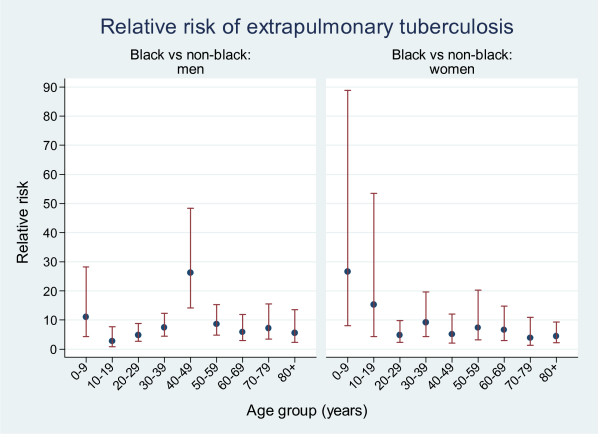
**Relative risk of extrapulmonary tuberculosis in men and women by race, 2000-2006**.

The incidence rates of tuberculosis stratified by sex, race, and site of disease are shown in Table 3. The incidence rate ratio of extrapulmonary tuberculosis in blacks compared to non-blacks was 5.5 (95% CI 4.7-6.5). The incidence rate ratio of extrapulmonary tuberculosis in men compared to women was 1.7 (95% CI 1.4-2.0). Black men had the highest annual rate of extrapulmonary tuberculosis (5.93 per 100,000 population), followed by black women (3.21 per 100,000), non-black men (1.01 per 100,000 population), and non-black women (0.58 per 100,000 population). Blacks had consistently higher rates of extrapulmonary tuberculosis compared to non-blacks at all ages, but the disparity was greatest in males aged 0-9 and 40-49 years, and in females aged 0-9 and 10-19 years (Figure 1).

**Table 3:** Incidence rates and relative risks of tuberculosis in men and women (per 100,000 population), 2000-2006 by race

	Black Men (pop. 2,984,932)		Non-Black Men (pop. 16,313,588)			Black Women (pop. 3,649,423)		Non-Black Women (pop. 17,001,005)		
	Cases^a^	Incidence^b^	Cases	Incidence	Relative Risk^c ^(95% CI)	Cases	Incidence	Cases	Incidence	Relative Risk^d ^(95% CI)
All Tuberculosis	630	21.11	784	4.81	4.39 (3.95-4.88)	303	8.30	399	2.35	3.54 (3.05-4.11)

Pulmonary Tuberculosis	453	15.18	619	3.79	4.00 (3.54-4.51)	186	5.10	301	1.77	2.88 (2.40-3.46)

Extrapulmonary Tuberculosis	177	5.93	165	1.01	5.86 (4.74-7.25)	117	3.21	98	0.58	5.56 (4.25-7.27)

^a ^Tuberculosis cases

^b^Incidence rate per 100,000 population

^c ^Incidence in black men/incidence in non-black men

^d ^Incidence in black women/incidence in non-black women

When the analysis was restricted to culture-confirmed extrapulmonary cases, black men still had the highest annual rate of extrapulmonary tuberculosis (3.97 per 100,000 population), followed by black women (1.92 per 100,000 population), non-black men (0.64 per 100,000 population), and non-black women (0.38 per 100,000 population).

### Proportion of extrapulmonary tuberculosis

The differences in the proportion of extrapulmonary disease stratified by race and sex are shown in Table 4. Among all persons with tuberculosis, blacks were more likely to present with extrapulmonary disease than non-blacks (31.5% vs. 22.2%, p < 0.001) and women were more likely to present with extrapulmonary disease than men (30.5% vs. 24.3%, p = 0.002). Black women were most likely to present with extrapulmonary disease (38.6%), followed by black men (28.1%), non-black women (24.6%), and non-black men (21.1%).

**Table 4:** Proportion of tuberculosis patients with extrapulmonary tuberculosis, Tennessee, 2000-2006, stratified by race

Site of Tuberculosis	Black Men (n=631)	Black Women (n=303)	p-value^a^	Non-Black Men (n=784)	Non-Black Women (n=399)	p-value^a^
Extrapulmonary	177 (28.1)	117 (38.6)	0.001	165 (21.1)	98 (24.6)	0.17

Lymphatic	57 (9.0)	64 (21.1)	< 0.001	33 (4.2)	25 (6.3)	0.12

**NOTE**. Data are presented as number(%) unless otherwise noted.

^a^Chi-square test.

When the analysis was restricted to culture-confirmed extrapulmonary cases, black women were still more likely to present with extrapulmonary disease (32.6%) compared to black men (23.3%), non-black women (20.2%) and non-black men (16.6%).

### Factors associated with extrapulmonary tuberculosis among persons with tuberculosis

Although a test for interaction between race and sex was not significant (p = 0.77), based on the different incidence and proportion of extrapulmonary disease by race and sex described above, we stratified our logistic regression analysis by these factors (Table 5). Black women were significantly more likely to have extrapulmonary tuberculosis than non-black men after adjustment for age, HIV infection, foreign birth, and socioeconomic factors including excess alcohol use, drug use, and homelessness. Black men also had higher odds of extrapulmonary tuberculosis than non-black men. HIV infection and foreign birth were independently associated with higher odds of extrapulmonary tuberculosis. Alcohol use was significantly associated with lower odds of extrapulmonary disease. Age over 20 years in 10-year increments was associated with lower odds of extrapulmonary disease, though this was not always statistically significant.

**Table 5:** Multivariable analysis of factors associated with extrapulmonary tuberculosis - persons with missing HIV status excluded.

Characteristic	Univariate Odds Ratio (95% CI)	p-value	Multivariate Odds Ratio (95% CI)	p-value
Non-Black Men	ref	n/a	Ref	n/a

Non-Black Women	1.22 (0.92-1.62)	0.17	1.09 (0.76-1.57)	0.62

Black Men	1.47 (1.15-1.87)	0.002	1.54 (1.13-2.09)	0.006

Black Women	2.35 (1.77-3.15)	< 0.001	1.82 (1.24-2.65)	0.002

HIV infection	1.35 (0.97-1.87)	0.07	1.45 (0.99-2.11)	0.06

Excess alcohol use	0.46 (0.35-0.61)	< 0.001	0.56 (0.39-0.80)	0.002

Foreign Birth	1.46 (1.15-1.85)	0.002	1.55 (1.12-2.14)	0.009

Injection drug use	1.03 (0.50-2.14)	0.93	1.32 (0.58-3.01)	0.50

Noninjection drug use	0.87 (0.62-1.20)	0.40	1.08 (0.72-1.62)	0.72

Homeless	0.67 (0.46-0.96)	0.03	0.93 (0.60-1.44)	0.74

Age (years)				

0-9	ref	n/a	ref	n/a

10-19	0.81 (0.43-1.53)	0.52	1.28 (0.51-3.22)	0.60

20-29	0.36 (0.22-0.58)	< 0.001	0.53 (0.25-1.11)	0.09

30-39	0.45 (0.28-0.72)	0.001	0.71 (0.34-1.49)	0.37

40-49	0.25 (0.15-0.40)	< 0.001	0.48 (0.22-1.01)	0.05

50-59	0.24 (0.14-0.39)	< 0.001	0.49 (0.23-1.05)	0.06

60-69	0.31 (0.18-0.51)	< 0.001	0.59 (0.27-1.32)	0.20

70-79	0.22 (0.13-0.38)	< 0.001	0.52 (0.23-1.18)	0.12

80+	0.30 (0.18-0.49)	< 0.001	0.87 (0.39-1.93)	0.73

With the exception of increasing age being associated with lower odds of extrapulmonary tuberculosis, the analysis using imputed HIV status for persons in whom it was missing had similar results to that with such patients (Table 6).

**Table 6:** Multivariable analysis of factors associated with extrapulmonary tuberculosis - HIV status imputed for persons with missing HIV status

Characteristic	Univariate Odds Ratio (95% CI)	p-value	Multivariate Odds Ratio (95% CI)	p-value
Non-Black Men	ref	n/a	Ref	n/a

Non-Black Women	1.22 (0.92-1.62)	0.17	1.07 (0.80-1.46)	0.63

Black Men	1.47 (1.15-1.87)	0.002	1.55 (1.18-2.03)	0.002

Black Women	2.35 (1.77-3.15)	< 0.001	1.93 (1.40-2.66)	< 0.001

HIV seropositivity	1.46 (1.06-2.00)	0.02	1.45 (0.99-2.14)	0.06

Excess alcohol use	0.46 (0.35-0.61)	< 0.001	0.53 (0.38-0.72)	< 0.001

Foreign Birth	1.46 (1.15-1.85)	0.002	1.50 (1.10-2.06)	0.011

Injection drug use	1.03 (0.50-2.14)	0.93	1.22 (0.46-3.23)	0.70

Noninjection drug use	0.87 (0.62-1.20)	0.40	1.11 (0.74-1.64)	0.62

Homeless	0.67 (0.46-0.96)	0.03	0.89 (0.58-1.37)	0.60

Age (years)				

0-9	ref	n/a	ref	n/a

10-19	0.81 (0.43-1.53)	0.52	0.76 (0.38-1.50)	0.42

20-29	0.36 (0.22-0.58)	< 0.001	0.36 (0.21-0.60)	< 0.001

30-39	0.45 (0.28-0.72)	0.001	0.47 (0.28-0.77)	0.003

40-49	0.25 (0.15-0.40)	< 0.001	0.34 (0.20-0.58)	< 0.001

50-59	0.24 (0.14-0.39)	< 0.001	0.32 (0.19-0.54)	< 0.001

60-69	0.31 (0.18-0.51)	< 0.001	0.44 (0.26-0.76)	0.004

70-79	0.22 (0.13-0.38)	< 0.001	0.32 (0.18-0.56)	< 0.001

80+	0.30 (0.18-0.49)	< 0.001	0.43 (0.25-0.72)	0.002

## Discussion

The incidence of a disease manifestation and the proportion of diseased persons with that manifestation are two different epidemiologic methods to assess the extent of a disease in a population. Incidence measures the risk of disease [17]. An individual's risk of extrapulmonary tuberculosis is determined by the degree of exposure to the pathogen and host immune factors that affect tuberculosis risk after *M. tuberculosis *infection (e.g., HIV infection). The proportion of extrapulmonary disease among tuberculosis patients is not likely associated with tuberculosis exposure; it is more indicative of the host's immune response to *M. tuberculosis*. Despite our extensive knowledge of tuberculosis epidemiology and human immunology, two questions remain unanswered: which factors determine whether an individual develops tuberculosis after *M. tuberculosis *infection; and which factors are associated with extrapulmonary dissemination? In this study, we sought to identify populations at highest risk of extrapulmonary tuberculosis.

We found in our study population that blacks had a significantly increased risk of extrapulmonary tuberculosis compared to non-blacks. This may be related to higher overall tuberculosis rates in blacks than non-blacks in the United States, in which the tuberculosis incidence is nearly 8-fold higher in U.S.-born blacks than U.S.-born whites [18]. Several factors likely contribute to this racial disparity, including differences in tuberculosis exposure, socioeconomic status, and access to medical care [19]. Another possible explanation is that the prevalence of HIV infection is higher in blacks and the resulting immunodeficiency leads to a higher risk of extrapulmonary tuberculosis.

There have been no recent studies that quantified the incidence of extrapulmonary tuberculosis in blacks. A study published in 1979 assessed the burden of tuberculosis in the United States by sampling thirteen states and two cities [10] and reported a 5-fold increase in rates of extrapulmonary TB in blacks compared to whites (6.5 vs 1.3 per 100,000 population). We found similar rates in blacks in our study. Interestingly, although men have traditionally had higher tuberculosis incidence rates than women, in our population, black women had higher incidence of extrapulmonary tuberculosis than non-black men (3.21 vs. 1.01 per 100,000 population). This suggests that black race may play a more important role in extrapulmonary tuberculosis than male sex.

We found that among all persons with tuberculosis, black women had the highest proportion of extrapulmonary disease (38.6%) compared to black men (28.1%), non-black women (24.6%), and non-black men (21.1%). Our results are consistent with previous findings that among persons with tuberculosis, blacks have a higher proportion of extrapulmonary disease compared to whites [7,19,20]. Our study is unique in that we stratified our findings by sex and discovered an interesting paradox: while black men had the highest incidence of extrapulmonary tuberculosis, among all persons who developed tuberculosis, black women, in addition to black men, had the highest odds of presenting with extrapulmonary disease after adjusting for HIV infection, foreign birth, as well as markers of low socioeconomic status such as excess alcohol use, drug use, and homelessness.

After infection with *M. tuberculosis*, the site of disease is likely determined by interactions between the pathogen and the host immune response. A recent study found that United States tuberculosis patients born in South Asian countries had higher rates of extrapulmonary tuberculosis compared to other foreign born patients, even after controlling for age and HIV co-infection [21]. This study suggested that South Asians may have an as-yet unidentified physiologic response to *M. tuberculosis *that increase their risk to develop disseminated disease. An intriguing hypothesis is that vitamin D deficiency, which is more common in dark-skinned individuals [22], predisposes to extrapulmonary tuberculosis. Both epidemiologic and laboratory-based studies have suggested a link between vitamin D deficiency and susceptibility to tuberculosis [23-25]. One study found that among Gujarati Asians living in London, the f allele of the *Fok1 *locus of the vitamin D receptor gene was associated with extrapulmonary tuberculosis [26].

While men typically have higher overall rates of tuberculosis compared with women [27], previous studies have shown that among persons who develop tuberculosis, women are more likely to have extrapulmonary tuberculosis than men [7,19,28-31]. A case-control study showed that 16.9% of female patients had extrapulmonary disease compared to 9.3% of male patients [7]. Similar findings have also been seen in Asian populations; 23.1% of women registered for tuberculosis treatment in Hong Kong had extrapulmonary tuberculosis compared to only 9.3% of men [30]. Ours is the first study that stratified results by race and discovered that black women in particular had high odds of extrapulmonary tuberculosis.

Our study had several limitations. We had a substantial number of tuberculosis cases with missing HIV status. Based on demographic characteristics, these persons were likely at low risk for HIV infection. This could explain why most of the tuberculosis cases with missing HIV status were not tested for HIV. Of note, the multivariable analysis that imputed HIV status for persons in whom it was missing had similar findings to the analysis in which such persons were excluded.

Approximately one-third of the extrapulmonary cases in our study were not culture-confirmed. Extrapulmonary tuberculosis is often paucibacillary, which decreases the sensitivity of AFB smears and cultures [32]. However, all cases of tuberculosis in our cohort were verified according to the Centers for Disease Control and Prevention surveillance definition [13]. In addition, we found similar results when restricting the analysis to culture-confirmed cases only.

While previous studies have found that mandatory reporting of tuberculosis cases to health departments is comprehensive [11], it is possible that not all cases of tuberculosis were reported during the study period. Failure to report cases would not be expected to differ based on race or sex and would be unlikely to affect study results.

Finally, several variables in our dataset, such as excess alcohol use and illicit drug use, were obtained via interview with the patient and may be inaccurate. However, previous work has shown that in the case of alcohol consumption, persons are more likely to underestimate, rather than overestimate their intake [33]. The database of tuberculosis cases did not include information on nutritional status, co-morbid conditions other than HIV status, or smoking; smoking has been identified as a risk factor for pulmonary and not extrapulmonary tuberculosis [34-36].

## Conclusion

Our study had important findings. Black men and black women had the highest incidence of extrapulmonary tuberculosis, and among all persons with tuberculosis, had the highest odds of presenting with extrapulmonary disease. Both findings support a hypothesis of an underlying predisposition to extrapulmonary tuberculosis in blacks, but the latter finding is particularly supportive because it is less related to tuberculosis exposure. Further understanding of risk factors that predispose to various forms of tuberculosis will allow for the development of strategies to prevent it and decrease its global burden. In addition, the high proportion of patients with missing HIV status highlights the importance of improving HIV testing in this high risk population [37].

## Competing interests

The authors declare that they have no competing interests.

## Authors' contributions

CTF and TRS were primarily responsible for study design, data analysis, and manuscript preparation. MRG contributed to study design and critical review of the manuscript. PGA and LK provided statistical support and critical review of the manuscript. JW and EH contributed to data acquisition. All authors read and approved the final manuscript.

## Pre-publication history

The pre-publication history for this paper can be accessed here:

http://www.biomedcentral.com/1471-2334/10/16/prepub

## Supplementary Material

Additional File 1 **Tennessee Demographic Information**. A brief paragraph describing basic demographic information about the state of Tennessee and the organization of the state health department. Click here for file
