# Supplementary material for: Black race, sex, and extrapulmonary tuberculosis risk: an observational study
Source: BMC Infect Dis. 2010 Jan 22;10:16. doi: 10.1186/1471-2334-10-16 (PMC2823615; doi:10.1186/1471-2334-10-16)
Supplement: Additional File 1 — Tennessee Demographic Information. A brief paragraph describing basic demographic information about the state of Tennessee and the organization of the state health department. [file 1471-2334-10-16-S1.DOC]

**Appendix – Tennessee Demographic Information**

The estimated population of Tennessee in 2008 was 6,144,104 persons. 48.7% of the population is male, 79.3% are white, 16.5% are black, 1.3% are Asian, and 3.5% are Latino. The median age is 37.5. The estimated number of foreign born persons is 246,050, or 4% of the population. The per capita income is $24,094 (2008 inflation adjusted dollars). The Tennessee public health system consists of a state Department of Health with the central office being located in the capital city of Nashville. Organizationally, the state system consists of 89 rural counties that are combined, regionally, into regional health offices. These rural county regions are components of the Tennessee Department of Health and are governed accordingly.

Reference: **US Census Bureau. Data Ferrett.** *Current Population Survey (Basic)*

June 2000-June 2006.
